# Development and validation of a prognostic model for kidney function 1 year after combined pancreas and kidney transplantation using pre-transplant donor and recipient variables

**DOI:** 10.1007/s00423-018-1712-z

**Published:** 2018-10-18

**Authors:** Katharina S. Zorn, Simon Littbarski, Ysabell Schwager, Alexander Kaltenborn, Jan Beneke, Jill Gwiasda, Thomas Becker, Felix Braun, Benedikt Reichert, Jürgen Klempnauer, Harald Schrem

**Affiliations:** 1 Core Facility Quality Management Transplantation, Integrated Research and Treatment Center Transplantation (IFB-Tx), Hannover Medical School, Hannover, Germany; 2 Trauma and Orthopedic Surgery, Federal Armed Forces Hospital Westerstede, Westerstede, Germany; 3 General, Visceral, Thoracic, Transplant and Pediatric Surgery, University Medical Center Schleswig-Holstein, Kiel, Germany; 4 Department of General, Visceral and Transplantation Surgery, Hanover Medical School, Carl-Neuberg-Str. 1, 30625 Hannover, Germany

**Keywords:** Prognostic scores, Simultaneous pancreas kidney transplantation, Donor variables, Recipient variables, Post-transplant graft function, Diabetic nephropathy

## Abstract

**Purpose:**

The widening gap between demand and supply of organs for transplantation provides extraordinary challenges for ethical donor organ allocation rules. The transplant community is forced to define favorable recipient/donor combinations for simultaneous kidney-pancreas transplantation. The aim of this study is the development of a prognostic model for the prediction of kidney function 1 year after simultaneous pancreas and kidney transplantation using pre-transplant donor and recipient variables with subsequent internal and external validation.

**Methods:**

Included were patients with end-stage renal failure due to diabetic nephropathy. Multivariable logistic regression modeling was applied for prognostic model design with retrospective data from Hannover Medical School, Germany (01.01.2000–31.12.2011) followed by prospective internal validation (01 Jan. 2012–31 Dec. 2015). Retrospective data from another German transplant center in Kiel was retrieved for external model validation via the initially derived logit link function.

**Results:**

The developed prognostic model is able to predict kidney graft function 1 year after transplantation ≥ KDIGO stage III with high areas under the receiver operating characteristic curve in the development cohort (0.943) as well as the internal (0.807) and external validation cohorts (0.784).

**Conclusion:**

The proposed validated model is a valuable tool to optimize present allocation rules with the goal to prevent transplant futility. It might be used to support donor organ acceptance decisions for individual recipients.

**Electronic supplementary material:**

The online version of this article (10.1007/s00423-018-1712-z) contains supplementary material, which is available to authorized users.

## Introduction

For patients suffering from diabetes and end-stage renal failure, simultaneous pancreas-kidney transplantation (SPK) is the best available therapeutic option leading to insulin independence and good kidney graft outcomes [1]. Kidney transplantation has become the gold standard for treating end-stage renal disease, prolonging patient survival and increasing quality of life compared to long-term dialysis [2]. Moreover, pancreas transplantation is a curative treatment option for type 1 diabetes that can lead to long-term insulin independence [3]. Successful pancreas transplantation enhances patients’ quality of life as insulin injections, and frequent controls of blood glucose concentrations are redundant [4]. Diabetes manifestations such as diabetic retinopathy, renal disease, or coronary heart disease are associated with significant morbidity and mortality [5]. Coronary heart disease accounts for 44% of fatalities in patients with type 1 diabetes and for 52% in patients with type 2 diabetes [6]. Under these considerations, SPK is a valuable therapeutic option as it cures diabetes and has beneficial effects on the progress of secondary complications [1].

Recently, survival rates 1-year after SPK were reported to be 96%, while 83% of the patients survived more than 5 years [7].

Due to increasing incidences of diabetes mellitus [8, 9], the demand for SPK will likely rise in the future [1, 10]. However, the gap between supply and demand of post-mortal organ donors is widening worldwide [11, 12], partly due to the repercussions of improved treatment strategies after traffic accidents and enhanced intensive care facilities [13]. Therefore, equitable organ allocation has been the focus of current research. This situation forces the transplant community to define characteristics determining appropriate pancreas and kidney donors and particularly favorable recipient/donor combinations for SPK. To ensure this, a reliable prognosis of outcome after SPK is an imminent necessity. Currently, no validated prognostic models are available predicting renal function 1 year after transplantation which is seen as an autonomous variable influencing long-term graft and patient survival [14]. Therefore, the current study aims to provide the first validated prognostic model for kidney function 1 year after SPK.

## Patients and methods

### Data collection

**Fig. 1:**
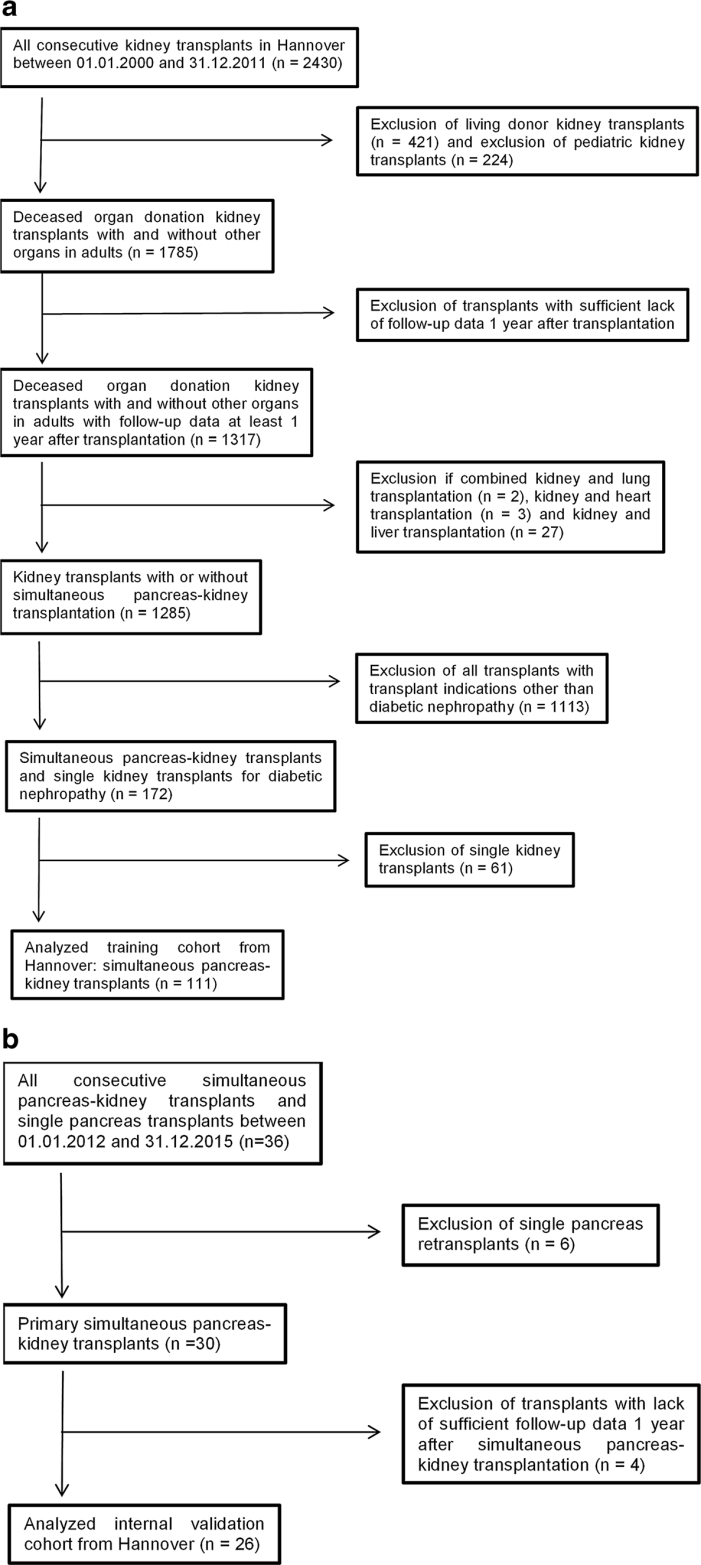
**a** Patient flow through the study for the training cohort from Hannover. **b** Patient flow through the study for the prospective internal validation cohort from Hannover

This is a retrospective observational analysis with data from routine databases from two German transplant centers (Hannover and Kiel) within the Eurotransplant community. Figure 1a, b demonstrates the patient flow through the study for the training and prospective validation cohorts from Hannover. After internal validation of the prognostic model, only those variables required for external validation were retrieved for 33 patients who underwent SPK in Kiel with a minimum follow-up of 1 year.

### Surgical procedures

All kidneys were transplanted into the left iliac fossa with subsequent secondary retroperitonalization using a transabdominal approach for the transplantation of both organs. All pancreas transplants were transplanted into the right fossa iliaca either with systemic venous or portal venous drainage and an arterial anastomosis to the common iliac artery. Exocrine drainage of the transplanted pancreas was realized by anastomosis of the grafts duodenum to the recipient’s ileum.

### Training cohort for prognostic model development

Included were simultaneous pancreas-kidney transplants for diabetic nephropathy after post-mortal organ donation between 01 Jan. 2000 and 31 Dec. 2011 in Hannover (*n* = 111). Pediatric patients (< 17 years) and those with follow-up after transplantation < 1 year were excluded from analysis.

### Internal validation cohort

Developed prognostic models were internally validated with data of 26 consecutive adult patients treated with SPK for diabetic nephropathy in Hannover between 01 Jan. 2012 and 31 Dec. 2015 (*n* = 26). The required minimal sample size for the validation cohorts was determined as 25 cases for a power of 0.80.

### External validation cohort

External validation of the developed prognostic model was performed using the data of 33 adult (> 17 years) consecutive patients from Kiel after SPK for diabetic nephropathy in Kiel (*n* = 33) between 01 Jun. 2008 and 31 Dec. 2015. Patients with lack of follow-up < 1 year after transplantation were excluded.

### Study end-points

The study end-point was kidney function kidney disease improving global outcomes (KDIGO) stage ≥ III after the first post-transplant year (± 6 weeks). Independent predictive factors for kidney function ≥KDIGO stage III 1 year after SPK were identified with univariable and multivariable logistic regression analyses. The results of these analyses were used to construct a model for the prediction of kidney function KDIGO stage ≥ III after the first post-transplant year (± 6 weeks). Glomerular filtration rate (GFR) categories (ml/min/1.73 m^2^) were used as described before to define KDIGO stages [15]. Patients with stage III ore more have a GFR < 59 (ml/min/1.73 m^2^).

The GFR was estimated as described by Levey et al. [16] as follows:

$$ {\displaystyle \begin{array}{c}\mathrm{GFR}\left[\mathrm{ml}/\min /1.73\ {\mathrm{m}}^2\right]={30849}^{\ast}\;\mathrm{standardized}\kern0.17em \mathrm{Serum}\kern0.37em \mathrm{Creatinine}{\left[\upmu \mathrm{mol}/\mathrm{l}\right]}^{-1.154}\ast \\ {}\mathrm{age}{\left[\mathrm{years}\right]}^{-0.203\ast}\left(1.212,\mathrm{if}\kern0.17em \mathrm{black}\right)\ast \left(0.742,\mathrm{if}\kern0.17em \mathrm{female}\right)\end{array}} $$

### Prognostic model design

Potential prognostic factors that are commonly known prior to transplantation were assessed using univariable binary logistic regression analyses with KDIGO stage ≥ III as response in the training cohort from Hannover (see Table 1).

**Table 1:** Shown are the preoperative recipient and donor variables determined directly prior to transplantation and their statistical influence on kidney function (KDIGO ≥III) 1 year after transplantation in the complete Hannover cohort as determined with univariate logistic regression analysis (all values rounded to three decimals). Purposeful selected variables with a *p* value ≤ 0.200 were analyzed in multivariable logistic regression after exclusion of collinearity in principal component analysis. *Abbreviations:* CI confidence interval, GFR glomerular filtration rate, SPK simultaneous pancreas-kidney transplantation, HbA1c glycosylated hemoglobin type A1c, HLA human leukocyte antigen, UC I_KI urgency code immunized kidney recipient, UC T_KI urgency code transplantable kidney recipient, ICU intensive care unit

Univariable logistic regression analysis: Training cohort Hannover Medical School Influences of pre-transplant recipient and donor variables on kidney-graft function (KDIGO ≥ III) 1 year after SPK				
Recipient variables	Continuous variables	*p* value	Odds ratio	95% CI
	Waiting time in months	0.855	0.998	0.973–1.025
	Age at SPK [years]	0.260	1.033	0.977–1.094
	Weight [kg]	0.136	1.031	0.991–1.076
	Height [cm]	0.007	1.072	1.019–1.133
	BMI [kg/m^2^]	0.697	0.971	0.838–1.127
	Duration of dialysis [months]	0.650	0.996	0.978–1.014
	Cold ischemic period [min] (kidney)	0.775	1.001	0.998–1.003
	Warm ischemic period [min] (kidney)	0.464	1.016	0.988–1.074
	HbA1c [%]	0.115	1.590	0.899–3.049
	Time from diabetes diagnosis to SPK [years]	0.381	1.025	0.971–1.084
	Binary variables	*p* value	Odds ratio	95% CI
	Male (yes)	0.007	3.298	1.395–8.006
	Death (yes)	0.491	1.750	0.418–11.946
	Blood group A (yes)	0.533	1.314	0.561–3.190
	Blood group B (yes)	0.893	1.120	0.241–7.948
	Blood group 0 (yes)	0.252	0.612	0.259–1.418
	UC T_KI (yes)	0.041	8.889	1.088–183.853
	UC I_KI (yes)	0.041	0.112	0.005–0.919
	Hyperparathyroidism (yes)	0.862	1.077	0.464–2.503
	Parathyroidectomy (yes)	0.189	4.085	0.732–76.648
	Pre-transplant Dialysis (yes)	0.722	1.375	0.184–7.454
	Insulin therapy after discharge (yes)	0.890	0.916	0.279–3.566
	Amputation (yes)	0.867	1.125	0.309–5.351
	Diabetic retinopathy (yes)	0.015	4.626	1.353–16.976
	Diabetic neuropathy (yes)	0.361	1.480	0.639–3.491
	Coronary heart disease (yes)	0.405	1.447	0.611–3.588
Donor variables	Continuous variables	*p* value	Odds ratio	95% CI
	Age [years]	< 0.001	1.079	1.039–1.125
	Weight [kg]	0.001	0.924	0.880–0.965
	Height [cm]	< 0.001	0.855	0.793–0.911
	BMI [kg/m^2^]	0.559	0.947	0.788–1.137
	Time of ventilation [h]	0.909	1.000	0.996–1.005
	Duration on ICU [h]	0.788	1.000	0.996–1.004
	GFR	0.575	0.004	−0.009 – 0.016
	Potassium [mmol/l]	0.931	0.967	0.444–2.106
	Urea [mmol/l]	0.693	1.030	0.894–1.210
	Number of HLA-A mismatches	0.893	1.043	0.564–1.959
	Number of HLA-B mismatches	0.065	0.437	0.174–1.022
	Number of HLA-DR mismatches	0.597	0.835	0.416–1.613
	Binary variables	*p* value	Odds ratio	95% CI
	Male (yes)	< 0.001	0.029	0.004–0.105
	Blood group A (yes)	0.534	1.314	0.561–3.190
	Blood group B (yes)	0.893	1.120	0.241–7.948
	Blood group 0 (yes)	0.254	0.612	0.259–1.418
	Blood group Rhesus positive (yes)	0.075	2.582	0.888–7.371
	Hypotensive periods (yes)	0.392	1.972	0.481–13.354
	Smoking (yes)	0.684	1.225	0.476–3.446
	Urine erythocytes (yes)	0.713	1.231	0.430–4.079

Multivariable principal component analysis was applied to avoid multi-collinearity in regression by choosing one of two variables in cases of high correlations (*R* ≥ |0.500|) between two variables. Principal component analyses and multivariable logistic regression analyses were performed separately for donor and recipient variables since they belong to distinct biological entities prior to transplantation [17].

Uncorrelated variables in principal component analysis (*R* < |0.500|) with *p* values ≤ 0.200 in univariable logistic regression analysis were included into multivariable logistic regression modeling using purposeful selection of co-variables after clinical judgment by the authors. Such an approach has been previously described by Hosmer, Lemeshow, and Sturdivant [18]. An initial stepwise backward likelihood elimination process of the least significant variables was followed by stepwise forward likelihood inclusion starting with the most significant variables which had been excluded before in order to identify potential variable interactions and to reach a preliminary multivariable logistic regression model. A threshold of > 20% change between each of the steps in one or multiple betas of the investigated variables was chosen for the anticipation of potentially significant factor interactions. Anticipated factor interactions prompted the creation of design interaction variables by multiplication of potentially interacting variables. These design variables were individually added to the preliminary multivariable model to check their independent significance using the effect-likelihood ratio test at conventional α-levels (*p* < 0.050). Only significant design variables were finally added to the final multivariable binary logistic regression model.

### Model validation

The prospective validation cohort from Hannover and the external retrospective validation cohort from Kiel were used for model validation in terms of sample validity and addressing possible center bias. These cohorts were also utilized for controlling ad hoc data-fitting.

### Statistical evaluation of derived prognostic models

This study was designed and executed in full accordance to the TRIPOD guidelines [19]. Prognostic model fit was assessed with the Hosmer-Lemeshow test to exclude potential model over fit. Evaluation of derived prognostic models was performed using receiver operating characteristic (ROC) curve analysis with determination of the area under the ROC curve (AUROC) for the prediction of KDIGO stage ≥ III 1 year after transplantation. Cut-off values were determined with the best Youden index (Youden index = sensitivity + specificity – 1) [20]. The Wilcoxon test, the Kruskal-Wallis test, and Kaplan-Meier analyses with log-rank tests as well as univariable linear regression were applied where appropriate.

JMP Pro statistics software version 11.2.0 (SAS Institute, Cary, NC, USA) was used to perform statistical tests. MedCalc Statistical Software, version 16.2.0 [21], was utilized for power and sample size calculations. The level of significance was defined as *p* < 0.050, except when indicated otherwise.

### Ethical considerations

The Ethics Committee at Hannover Medical School has reviewed and approved this study (Approval Number: 3149-2016).

## Results

### The combined cohorts from Hannover for model training and validation (*n* = 137)

**Fig. 2:**
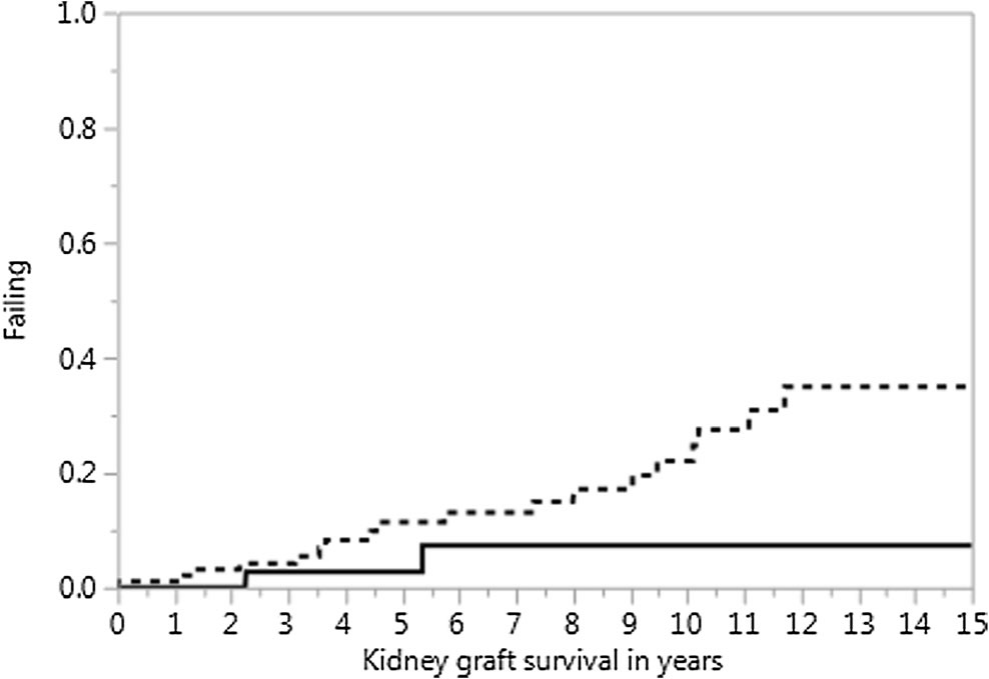
Shown is the influence of kidney graft function after the first year classified as KDIGO stage ≥ III on long-term kidney graft survival limited by all-cause graft failure in the combined development and internal validation cohorts from Hannover (*p* < 0.001, log-rank test)

Renal function 1 year after SPK classified as KDIGO ≥III had a significant influence on long-term all-cause graft loss after transplantation (*p* < 0.001, log-rank test) underlining the significance of the study-endpoint chosen for prognostic model development (Fig. 2).

Of all patients, 61.3% received tacrolimus-based immunosuppressive regimen and the remainder a cyclosporine-based protocol, each complemented with prednisolone. Of patients, 91.0% received additionally mycophenolate mofetil.

Kaplan-Meier estimates showed overall survival rates of 99.3%, 97.8%, and 95.9% one, three, and five years after SPK patients in the combined cohorts from Hannover. Regarding kidney graft survival, 98.5%, 96.3%, and 91.1% was still functional at 1, 3, and 5 years after SPK.

### Clinical outcome 1 year after SPK in the training cohort

Clinical and demographic characteristics of donors and recipients in the training cohort are summarized in Supplementary Tables 1 and 2. Median GFR 1 year after SPK was 49 ml/min. Renal graft function 1 year after SPK was assessed as KDIGO-stage I in 5 patients (4.5%), KDIGO-stage II in 25 patients (22.5%), KDIGO-stage III in 61 patients (55.0%), KDIGO-stage IV in 18 patients (16.2%), and KDIGO-stage V in 2 patients (1.8%). Pancreas graft failure was observed in 24 patients (21.8%) during long-term follow-up. Gender mismatch was observed in 52 cases (50.5%).

### Selection process of candidate variables for prognostic model design

The results of univariable logistic regression analysis are shown in Table 1. The following variables were revealed as significant risk factors for impaired kidney function 1 year post-transplant in univariable regression analysis: recipient height, male recipient, donor age, donor weight, donor height, and male donor (Table 1). Donor causes of death were excluded from analysis and the design of the first multivariable model due to a high percentage of missing values (49.5%).

### Prognostic model development with the training group from Hannover

All developed and investigated models (recipient model, donor model, final meta model) had no significant lack-of-fit test results (*p* > 0.050). The final prognostic model is a meta-model including the logits of the two separately developed models for recipient and donor factors.

A significant factor interaction was discovered for recipient age and the time from diabetes diagnosis until SPK, as well as between donor-GFR and donor-urea. Additional analyses revealed that the associations between the recipient age at SPK in quartiles as well as the time from diabetes diagnosis to SPK in quartiles and their respective parameter estimates with kidney graft function ≥ KDIGO III 1 year after SPK were non-linear as is shown in Supplementary Figs. 1 and 2. The best possible parameterization of age at SPK and time between diabetes diagnosis and SPK in the final prognostic model could be achieved by leaving both of these variables in the final prognostic model together with the independently significant design variable which describes the identified multiplicative interaction between these two factors. This was done irrespective of the fact that the variable recipient age at SPK alone had no significant independent influence (*p* = 0.084) on kidney graft function ≥ KDIGO III 1 year after SPK.

The logit of the multivariable recipient model and the logit of the multivariable donor model demonstrated a significant influence on kidney graft function (KDIGO ≥ III) 1 year after SPK. The logits of these two models were used as inputs for the final regression meta-model, which was also shown to have a significant influence on kidney function (KDIGO ≥ III) 1 year after SPK (Table 2).

**Table 2:** Shown are the influences of pre-transplant recipient and donor variables on kidney graft function (KDIGO ≥ III) 1 year after SPK as identified in the multivariable logistic regression model of recipient and donor risk factors for kidney function (all values rounded to three decimals). Data on the time from diabetes diagnosis to SPK [years] was missing for 14 patients. These cases were therefore excluded from the development of the model. CI confidence interval, *SPK* simultaneous pancreas-kidney transplantation, *GFR* glomerular filtration rate

Multivariable logistic regression analysis Training cohort Hannover Medical School Influences of pre-transplant recipient and donor variables on kidney-graft function (KDIGO ≥ III) 1 year after SPK				
Final model	Variables	*p* value	Odds ratio	95% CI
	Logit recipient model	0.001	4.975	2.077–15.385
	Logit donor model	< 0.001	2.889	1.878–5.321
Final recipient model	Recipient variables	*p* value	Odds ratio	95% CI
	Male (yes)	0.002	4.543	1.699–12.972
	Age at SPK [years]	0.084	0.816	0.616–1.025
	Time from diabetes diagnosis to SPK [years]	0.036	0.664	0.406–0.975
	Diabetic retinopathy (yes)	0.007	7.384	1.710–37.102
	Recipient age * time from diabetes diagnosis to SPK [years]	0.028	1.010	1.001–1.022
Final donor model	Donor variables	*p* value	Odds ratio	95% CI
	Male (yes)	< 0.001	1.812	1.082–2.803
	Age [years]	0.014	0.065	0.013–0.122
	Number of HLA-B mismatches	0.010	1.479	− 2.788 to − 0.341
	Donor GFR * donor urea	0.037	0.002	0.001–0.005

The final recipient model has the following equation:

$$ {\displaystyle \begin{array}{c}\mathrm{y}1=8.2688+\left(-{0.2000}^{\ast}\;\mathrm{Recipient}\ \mathrm{age}\right)+\\ {}\left(-{0.4103}^{\ast}\;\mathrm{Time}\kern0.17em \mathrm{from}\kern0.17em \mathrm{diabetes}\kern0.17em \mathrm{diagnosis}\kern0.17em \mathrm{until}\;\mathrm{SPK}\;\mathrm{in}\kern0.17em \mathrm{years}\right)+\\ {}\ \left(\mathrm{Diabetic}\kern0.17em \mathrm{retinopathy}\ \mathrm{if}\ \mathrm{y}\mathrm{es}=0.9997,\mathrm{if}\ \mathrm{no}=-0.9997\right)+\\ {}\left(\mathrm{Recipient}\kern0.17em \mathrm{male}\ \mathrm{if}\ \mathrm{y}\mathrm{es}=0.7568,\mathrm{if}\ \mathrm{no}=-0.7568\right)+\\ {}\left({0.0098}^{\ast}\;\left(\mathrm{Recipient}\;{\mathrm{age}}^{\ast}\;\mathrm{Time}\kern0.17em \mathrm{from}\ \mathrm{diabetes}\ \mathrm{diagnosis}\ \mathrm{until}\ \mathrm{SPK}\ \mathrm{in}\ \mathrm{years}\right)\right)\end{array}} $$

The following equation reflects the final donor model:

$$ {\displaystyle \begin{array}{c}\mathrm{y}2=0.8725+\left(\mathrm{Donor}\kern0.17em \mathrm{male}\ \mathrm{if}\ \mathrm{y}\mathrm{es}=-1.8123,\mathrm{if}\ \mathrm{no}=1.8123\right)+\\ {}\left({0.0650}^{\ast}\;\mathrm{Donor}\;\mathrm{age}\ \mathrm{in}\ \mathrm{years}\right)+\\ {}\left(-{1.4789}^{\ast}\;\mathrm{Number}\kern0.17em \mathrm{of}\ \mathrm{HLA}-\mathrm{B}\ \mathrm{mismatches}\right)+\\ {}\left({0.0023}^{\ast}\;\mathrm{Donor}\;{\mathrm{GFR}}^{\ast}\;\mathrm{Donor}\kern0.17em \mathrm{urea}\ \mathrm{in}\ \mathrm{mmol}/\mathrm{l}\right)\end{array}} $$

The equation of the finally developed prognostic meta-model was derived as

Probability of kidney function (KDIGO ≥ III) 1 year after SPK (%) = $\frac{1}{1-\exp (y3.)}$

$$ {\displaystyle \begin{array}{c}\mathrm{with}\\ {}\mathrm{y}3=-1.3289+\left({1.3354}^{\ast }\ \mathrm{y}1+{1.0980}^{\ast }\ \mathrm{y}2\right)\end{array}} $$

**Fig. 3:**
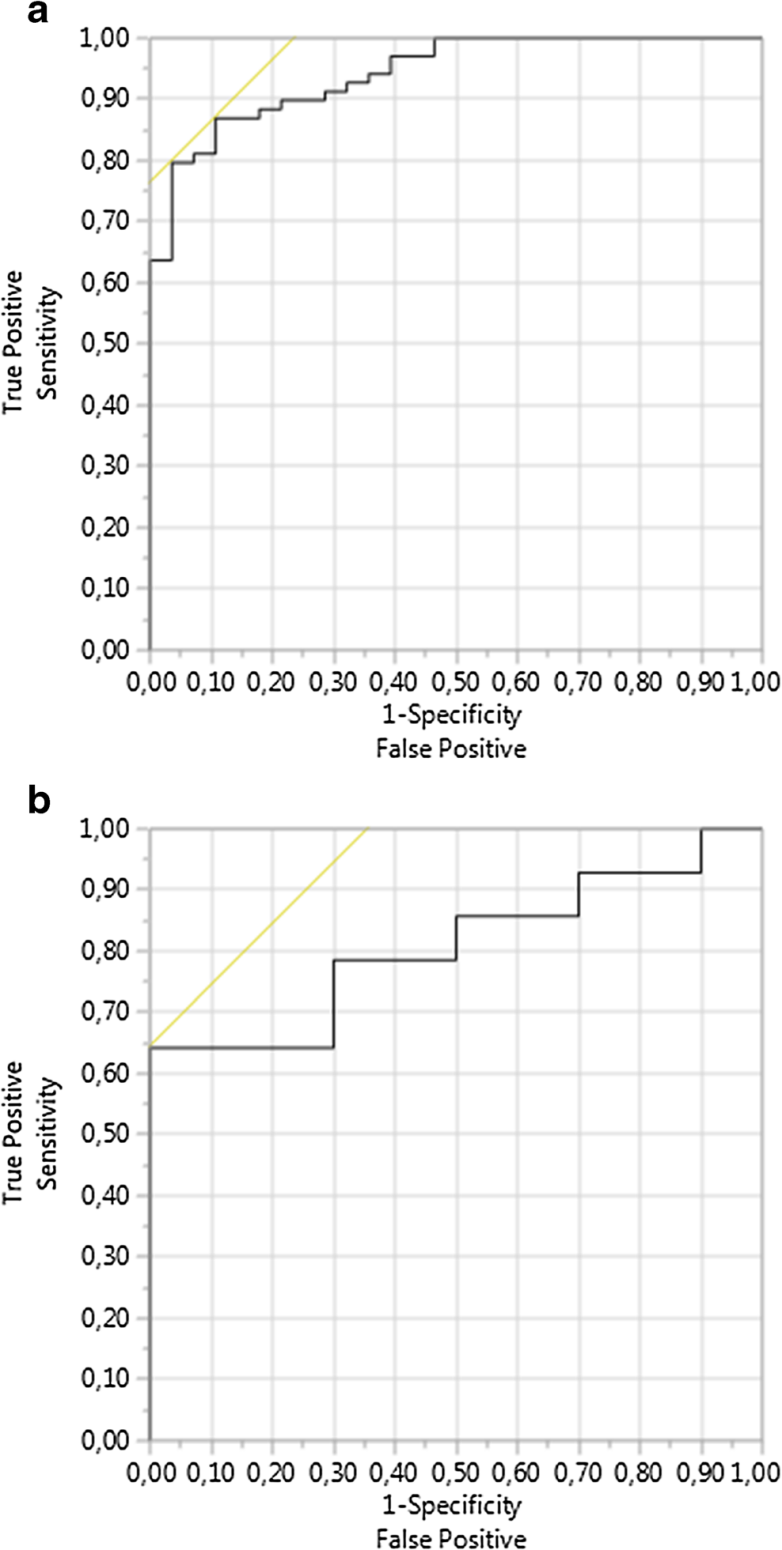
**a** Shown are the results of receiver operating characteristic (ROC) curve analysis of the final prognostic meta model for the prediction of the kidney function (KDIGO ≥ III) 1 year after SPK in the training cohort with an area under the ROC curve (AUROC) of 0.943.**b** Shown are the results of ROC curve analysis of the final prognostic meta model for the prediction of the kidney function (KDIGO ≥ III) 1 year after SPK in the internal prospective validation cohort with an Area under the ROC curve (AUROC) of 0.807 from Hannover

The ROC curves of the final prognostic meta-model in the training cohort and the validation cohorts are shown in Fig. 3. The area under the ROC curve (AUROC) was 0.943 in the training cohort from Hannover (Fig. 3a). The cut-off value for the prediction of the kidney function (KDIGO ≥ III) 1 year after SPK with the best sensitivity and specificity was determined using the best Youden-Index at a risk of 87.1% equaling a logit value of 0.791. Patients with logits larger than 0.791 have a significant risk of kidney function KDIGO stage ≥ III 1 year after SPK. The sensitivity, specificity, and overall correctness of this model were determined in the training cohort as 87.1%, 92.0%, and 89.55%, respectively.

The predicted probabilities of renal graft function KDIGO ≥ III 1 year after SPK using the proposed prognostic model with pre-transplant donor and recipient data versus actually observed KDIGO stages after 1 year are shown in Fig. 5.

### Prognostic model validation

The pre- and post-transplant characteristics of donor and recipient variables of the analyzed training, internal validation and external validation cohorts, are summarized in Table 3. The AUROC for the prediction of kidney function (KDIGO ≥ III) 1 year after SPK was 0.807 in the prospective internal validation cohort. The best sensitivity and specificity was determined in the internal validation cohort using the best Youden Index at a risk of 64.3% equaling a logit value of 0.543. The sensitivity, specificity, and overall correctness of this model in the internal validation cohort were determined as 64.3%, 90.1%, and 77.2%, respectively. The corresponding ROC curve is shown in Fig. 3b.

**Table 3:** Shown is the distribution of pre-operative recipient and donor variables of the internal validation cohort from Hannover and the validation cohort from Kiel (all variables rounded to two decimals). *GFR* glomerular filtration rate, *SPK* simultaneous pancreas-kidney transplantation, *HbA1c* glycosylated hemoglobin type A1c

	Internal training cohort Hannover		Internal validation cohort Hannover		External validation cohort Kiel
Pre-operative recipient variables					
Continuous variables	Median (min–max)	*p* value^a^	Median (min–max)	*p* value^b^	Median (min–max)
Age at SPK [years]	43 (23–63)	0.446	43 (27–55)	0.319	44 (28–57)
BMI [kg/m^2^]	24 (15–31)	0.412	24 (16–31)	0.379	23.3 (18.8–33.3)
Time from diabetes diagnosis to SPK [years]	28 (6–53)	0.487	31 (13–44)	0.007	21 (5–45)
HbA1c [%]	6 (4.6–10)	0.0096	5 (4.4–9.7)	< 0.001	7.9 (5.7–11.8)
Binary variables	*n* (% of cohort)	*p* value^a^	*n* (% of cohort)		*n* (% of cohort)
Male (yes)	71 (63.9%)	0.098	12 (46.15%)	0.726	20 (60.6%)
Diabetic retinopathy (yes)	99 (89.19%)	0.626	24 (92.31%)	0.459	27 (84.4%)
Post-operative recipient variables					
Binary variables	*n* (% of cohort)	*p* value^a^	*n* (% of cohort)		*n* (% of cohort)
KDIGO I (yes)	5 (4.5%)	0.143	0 (0%)	0.710	1 (3.1%)
KDIGO II (yes)	25 (22.5%)	0.047	11(42.31%)	0.110	12 (36.4%)
KDIGO III (yes)	61 (55%)	0.649	13 (50%)	0.967	18 (54.6%)
KDIGO IV (yes)	18 (16.2%)	0.237	2 (7.69%)	0.049	1 (3.1%)
KDIGO V (yes)	2 (1.8%)	0.357	0 (0%)	0.664	1 (3.1%)
KDIGO ≥ III (yes)	81 (73%)	0.135	15 (57.69%)	0.173	20 (60.6%)
Pre-operative donor variables					
Continuous variables	Median (min–max)	*p* value^a^	Median (min–max)		Median (min–max)
Age [years]	37 (11–51)	0.203	30.5 (13–47)	0.912	36 (12–53)
GFR	102.77 (20.12–235.15)	0.348	110.88 (58.50–264.08)	0.983	106.79 (38.59–286.84)
Urea [mmol/l]	3.8 (0.7–17.8)	0.039	3.2 (1.1–7)	0.482	3.5 (1–23)
Number of HLA-B mismatches	2 (0–2)	0.927	2 (1–2)	0.013	2 (1–2)

^a^Results of univariable logistic regression analysis for continuous variables and chi^2^ (Pearson) test for binary variables comparing the training cohort from Hannover and the internal validation cohort from Hannover

^b^Results of univariable logistic regression analysis for continuous variables and chi^2^ (Pearson) test for binary variables comparing the training cohort from Hannover and the external validation cohort from Kiel

**Fig. 4:**
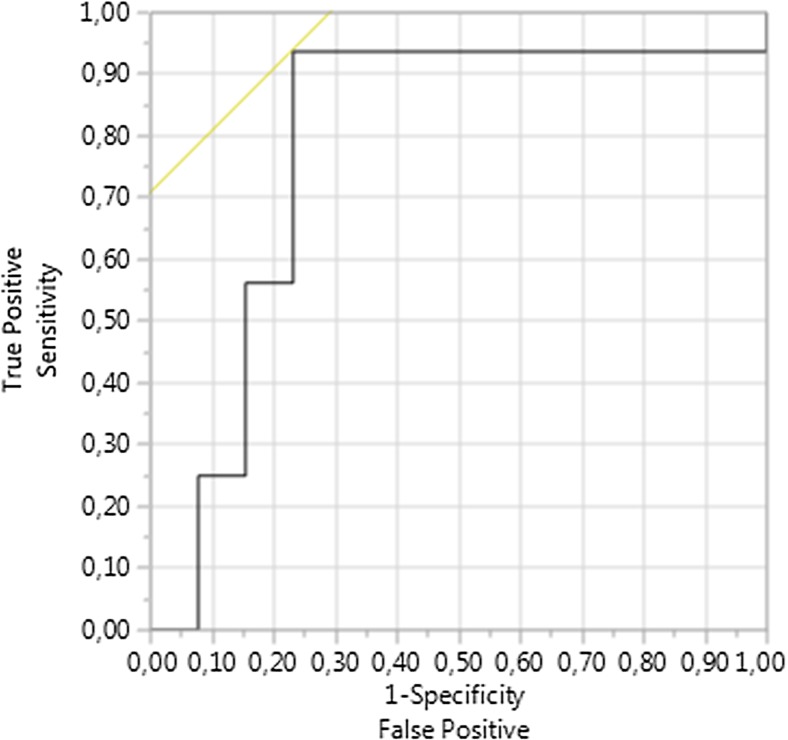
Shown are the results of ROC curve analysis of the final prognostic meta model for the prediction of the kidney function (KDIGO ≥ III) 1 year after SPK in the training cohort with an AUROC of 0.784 for the external retrospective validation cohort from Kiel

**Fig. 5:**
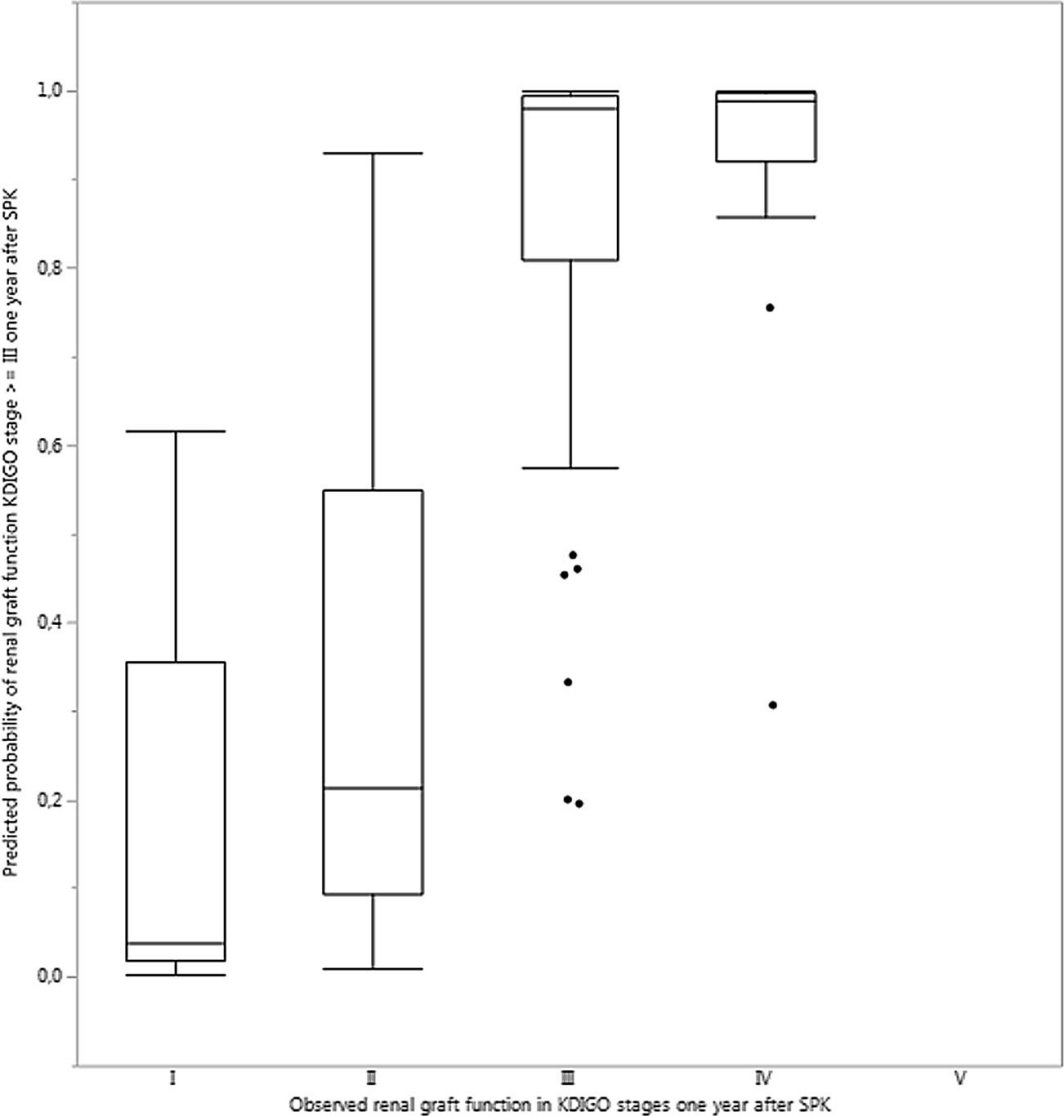
Shown are the predicted probabilities of renal graft function KDIGO ≥ III 1 year after SPK using the proposed prognostic model with pre-transplant donor and recipient data versus actually observed KDIGO stages after 1 year

External model validation revealed an AUROC for the forecast of impaired kidney function (KDIGO ≥ III) 1 year after SPK of 0.784 in Kiel. Figures 4 shows the associated ROC curve. Figures 5 shows predicted probabilities of renal graft function versus actually observed renal graft function according to the KDIGO stages 1 year after SPK using the proposed prognostic model in the training cohort. The best sensitivity and specificity was determined in the external validation cohort using the best Youden Index at a risk of 54.2% equaling a logit value of − 1.259. The sensitivity, specificity, and overall correctness of this model in the external validation cohort were determined as 87.5%, 84.6%, and 86.1%, respectively.

## Discussion

This study provides for the first time an internally and externally validated prognostic model for the prediction of impaired kidney graft function with a GFR < 60 (ml/min/1.73 m^2^) equaling KDIGO stage ≥III 1 year after SPK with pre-transplant donor and recipient data. As the gap between the need of organs and the supply of donor organs is widening [11, 12], this is a highly important finding as it provides a tool to avoid foreseeable futility of transplantation as a result of suboptimal donor and recipient combinations. The results of this study show that older male recipients with a longer time between diabetes diagnosis until transplantation suffering from diabetic retinopathy carry the highest risk of poor kidney graft function 1 year after SPK (KDIGO stage ≥ III) while male donors reduce this risk. This observation points to a possible advantage of transplanting male kidneys into male recipients. The validated prognostic model shows that the male sex of the recipient is an independently significant risk factor for kidney graft function ≥ KDIGO III 1 year after SPK while the male sex of the organ donor is an independently significant protective factor. Therefore, physicians could accept only male donor organs for male recipients with the goal to offset this identified risk associated with a male recipient sex. This study further shows that older donors with compromised kidney function as expressed by their higher urea and GFR values increase the risk of poor graft function after 1 year. The latter combination of donor factors should be avoided for recipients with high risk profiles. The relevance of the chosen study-endpoint defined as poor kidney graft function 1 year after SPK (KDIGO stage ≥ III) is supported by the fact that this end-point significantly decreased long-term graft survival in this study (Fig. 2). The results of this investigation are meaningful for the discussion of expected outcomes with patients and for the selection of donors for specific recipients.

A wider application of the proposed model might significantly improve donor allocation rules leading to superior transplant results. However, prior to introduction of this model into donor allocation rules in different health care systems, an assessment of the prognostic capability of the proposed model in the respective populations is highly recommendable as a recalibration of the model may be required for different populations. In this context, the recently published guidelines for the development and validation of prognostic models in medicine, the TRIPOD guidelines could be helpful [19]. These guidelines should be considered for future validation studies or recalibrations of the proposed model.

We believe that the remarkable reduction over 10% in the observed rates of KDIGO stage ≥ III in the internal and external validation cohorts as compared to the training cohort (Table 3) is a direct consequence of improvements in immunosuppression and perioperative care after 2012. These observations underline the very notable fact that the developed prognostic model could still be validated internally and externally despite these differences.

It is not surprising that pre-operative recipient variables do not have an exclusive influence on post-transplant kidney function 1 year after SPK. This study shows that pre-operative donor variables are also relevant for the prediction of kidney graft function after 1 year. Legendre and co-workers recently came to a similar intuitive conclusion [22].

This study clearly shows that the time between diabetes diagnosis and SPK, recipient age at transplantation, and pre-transplant diabetic retinopathy in the recipient have independent significant effects on kidney function 1 year after SPK. Diabetes with complications, such as diabetic retinopathy, obviously increases the risk of unfavorable kidney graft function after the first post-transplant year. This is especially the case in older recipients as expressed by the significant interaction variable detected in this study (recipient age * time from diabetes diagnosis to SPK), which is incorporated in the derived prognostic model. This is most likely due to the detrimental systemic effects of prolonged diabetes in older patients on the cardiovascular system, which is required for sufficient graft perfusion [23].

It is interesting to note that the time between diagnosis of diabetes and SPK and recipient age at transplantation were revealed as independent protective factors against impaired kidney function 1 year after transplantation while the multiplication of recipient age with the time between diagnosis of diabetes and SPK represents a significant independent interaction risk factor for impaired kidney graft function (Table 2). This observation may be partly due to the observed non-linear univariable influences of both of these variables on kidney graft function ≥ KDIGO III 1 year after SPK (Supplementary Figs. 1 and 2). The best possible parameterization of age at SPK and time between diabetes diagnosis and SPK in the final prognostic model could be achieved by leaving both of these variables in the final prognostic model together with the independently significant design variable which describes the identified multiplicative interaction between these factors. This was done following the suggested methodological approach which has been proposed before for such situations by Hosmer, Lemeshow, and Sturdivant [18]. Further analyses in the Hannover training cohort revealed that diabetic complications such as amputations and coronary heart disease were significantly more frequent in older patients (*p* = 0.008 and *p* < 0.001, respectively; Kruskal-Wallis test). Perhaps unsurprisingly so, additional univariable linear regression revealed that recipient age had a significant increasing influence on time between diabetes diagnosis and SPK (*p* < 0.001) while both factors demonstrated lack of strong correlation in principal component analysis (*R* = 0.459). The prognostic model that was developed and validated in this study appears to be able to differentiate between different risk profiles of older versus younger recipients by taking the recipient age combined with the time between diagnosis and SPK into account with an independent significant interaction variable. A further argument in favor of this interpretation is the fact that diabetic retinopathy has also been identified as an independent significant recipient risk factor for impaired kidney function 1 year after SPK.

The current study reveals that both donor and recipient sex are independent factors affecting kidney function 1 year after SPK. While male recipient sex increases the risk of impaired kidney function 1 year after SPK, male donor sex is a protective risk factor. Puoti et al. made this observation before [24]. Male recipients often show less concern with their graft and have a higher risk of diseases like ischemic heart disease and hypertension, which may affect kidney function. Their outcome might also be inferior due to compromised compliance to immunosuppressive therapy and lower estradiol levels in contrast to women, which could improve graft function [24]. On the other hand, diverse clinical studies have shown that female donor sex is a risk factor for shorter patient survival after SPK [25]. Kidney function is significantly better in recipients receiving organs from male donors [26]. This may also be due to increased number of nephrons in male kidneys or because of their smaller sensitivity for nephrotoxic impacts of some immunosuppressants, when compared to women [24]. It is widely known that different immune responses, hormonal settings, and metabolic conditions are related to the patient’s sex. Nevertheless, there is still a controversy about the role of sex in kidney transplantation and especially the role of gender mismatch as significant risk factor influencing graft survival, as different studies recommend different gender combinations for favorable outcomes [27].

It is not surprising that elderly donors are associated with worse graft function. Serum creatinine has been reported to be significantly higher while graft loss 1 year after transplantation is significantly more frequent in patients receiving organs from older donors [28]. The population of patients with end-stage renal disease is aging [29]. Studies have shown that kidney transplantation has a beneficial effect on patient survival in comparison to staying on dialysis [30]. Rao et al. made the observation that recipients older than 70 years had a 41% lower risk of mortality when compared to those who remained wait-listed [31]. For this reason, the Eurotransplant-Senior Program was developed to match donor-to-recipient age. Studies showed that there were no significant differences between patients who were matched by age and patients who received organs from younger donors [32]. Cohesive to this observation, a focus on donor-to-recipient age matching is warranted.

Up to six human leukocyte antigen (HLA) mismatches (2 HLA-A, 2 HLA-B, and 2 HLA-DR mismatches) were accepted for transplantation at Hannover Medical School. It is widely known that HLA matching results in improved outcomes after kidney transplantation [33]. However, the importance of HLA mismatches especially in SPK still remains controversial [34]. Lo et al. found that the number of HLA-A mismatches were associated with an increased risk of acute rejection in SPK patients [34]. In contrast, Rudolph and colleagues described that HLA mismatch has no significant influence on acute rejection [33]. It was observed that the number of HLA-B mismatches increases the risk of acute rejection in single pancreas-transplanted patients. However, the number of HLA-B mismatches could not be significantly associated with an increased risk of acute rejection in patients after SPK [33].

Interestingly, in the current study, neither HLA-A nor HLA-DR mismatches had an influence on kidney graft function 1 year after SPK. Furthermore, this study revealed that increasing numbers of HLA-B mismatches were an independent and significant protective factor for kidney function 1 year after SPK.

The observed associations of HLA-B mismatches with kidney function after SPK might well be the results of epigenetic phenomena, which are not yet fully understood. Fifty percent of the heredity of diabetes is conditioned by the HLA phenotype, especially HLA-DR/DQ haplotypes. In patients with diabetes type 1, there is a 90% chance to find one of these two haplotypes [35]. Additionally, there is recent evidence which connects specific HLA-B alleles with HLA-DR/DQ haplotypes which are known to play a relevant role in the development of diabetes [36]. Therefore, the protective effect of the number of HLA-B mismatches for kidney graft function may be associated with a decreased likelihood of diabetes recurrence in the transplanted pancreas which may protect kidney graft function.

Taken together, a clear and concise prognostic model was developed which incorporates recipient and donor variables. All included data is readily available in routine clinical practice. Strikingly, the developed prognostic model could be successfully validated in a prospective internal as well as an external validation cohort.

The proposed prognostic model for kidney function 1 year after SPK is of high relevance because recipients compete with other potential recipients of single kidney grafts in times of ubiquitous organ shortage. Futile transplantation could potentially be limited by adoption of the proposed prognostic model to current allocation rules.

There are limitations which need to be considered. Firstly, the developed prognostic model is limited by its validation within one country and healthcare system. Implementation of the model in other countries and populations cannot be assumed to be successful without further evaluating studies, which may indicate a need for model recalibration. Moreover, the proposed model has only been designed to predict kidney graft function 1 year after SPK. It does not predict pancreas graft function as required variables were not available in the current study’s database. Future studies should also consider the role of HLA antibody levels in the recipient determined for example with luminex technology, as these levels may be another relevant factor for the prediction of kidney graft function ≥ KDIGO III 1 year after SPK.

Due to a significant lack of data regarding the donor cause of death for nearly half of the analyzed cohort, it was unfortunately not possible to investigate its influence on the study endpoint. It is possible that this might be of relevance. However, since the presented model is able to predict the study endpoint reliably with pre-operatively available data, this study’s results might play an important role for future improvement in allocation of donor organs. The possible influence of donor cause of death is still a matter of debate and should be addressed systematically in further studies.

Further limitations might be that there was no detailed analysis undertaken regarding post-operative complications and the immunosuppressive regimen after transplantation. The influences of nephrotoxic post-transplant immunosuppression, immunological rejection, and post-operative complications on post-operative kidney graft function are well established [37]. Nevertheless, it is still striking that the proposed model could be internally and externally validated in its ability to predict outcome and it needs to be stressed that only pre-operatively available data was used for this prediction model.

The promising results of this study should be evaluated in future trials with higher evidence levels, ideally focusing on further external evaluation in other countries. This could potentially optimize allocation schemes with regard to successful outcome 1 year after SPK.

## Electronic supplementary material

ESM 1 (DOCX 27 kb)

ESM 2 (DOCX 27 kb)

ESM 3 (DOCX 21 kb)

ESM 4 (DOCX 19 kb)
