# Supplementary material for: Development and validation of a prognostic model for kidney function 1 year after combined pancreas and kidney transplantation using pre-transplant donor and recipient variables
Source: Langenbecks Arch Surg. 2018 Oct 18;403(7):837–49. doi: 10.1007/s00423-018-1712-z (PMC6244698; doi:10.1007/s00423-018-1712-z)
Supplement: Supplementary file 2 — (DOCX 27 kb) [file 423_2018_1712_MOESM2_ESM.docx]

**Supplementary Figure 2**: Shown is the non-linear association between the midpoints of time from diabetes diagnosis to SPK (years) in quartiles and their respective parameter estimates for their influences on kidney graft function ≥ KDIGO III one year after SPK.

**Title: Development and validation of a prognostic model for kidney function one year after combined pancreas and kidney transplantation using pre-transplant donor and recipient variables**

Journal Name: Langenbeck’s Archives of Surgery

Authors: Katharina S. Zorn, Simon Littbarski , Ysabell Schwager , Alexander Kaltenborn, Jan Beneke, Jill Gwiasda , Thomas Becker, Felix Braun, Benedikt Reichert, Jürgen Klempnauer, Viktor Arelin, Harald Schrem

Corresponding Author: Harald Schrem, MD; [schrem.harald@mh-hannover.de](mailto:schrem.harald@mh-hannover.de)

Affiliations:

Core Facility Quality Management Transplantation, Integrated Research and Treatment Center Transplantation (IFB-Tx), Hannover Medical School, Hannover, Germany

Department of General, Visceral and Transplantation Surgery, Hanover Medical School, Hannover, Germany
