# Supplementary material for: Development and validation of a prognostic model for kidney function 1 year after combined pancreas and kidney transplantation using pre-transplant donor and recipient variables
Source: Langenbecks Arch Surg. 2018 Oct 18;403(7):837–49. doi: 10.1007/s00423-018-1712-z (PMC6244698; doi:10.1007/s00423-018-1712-z)
Supplement: Supplementary file 3 — (DOCX 21 kb) [file 423_2018_1712_MOESM3_ESM.docx]

|  | **Training cohort Hannover Medical School (n = 111)** | | |
| --- | --- | --- | --- |
| **Pre-operative recipient variables** | **Continuous Variables** | **median (min – max)** | **missing values** |
|  | Waiting time in months | 15 (0 – 77) | 0 |
|  | Age at SPK in years | 43 (23 – 63) | 0 |
|  | Weight [kg] | 71.5 (50 – 103) | 0 |
|  | Height [cm] | 173 (148 – 193) | n = 1 (0.9%) |
|  | BMI [kg/m²] | 24 (15 – 31) | n = 1 (0.9%) |
|  | Duration of dialysis [months] | 29 (0 – 123.4) | n = 1 (0.9%) |
|  | Time from diabetes diagnosis to SPK [years] | 28 (6 – 53) | n = 14 (12.6%) |
|  | HbA1c | 6 (4.6 – 10) | n = 2 (1.8%) |
|  | **Binary variables** | **n (% of cohort)** | **missing values** |
|  | Primary kidney transplantation (yes) | 107 (96.4%) | 0 |
|  | Second kidney transplantation (yes) | 4 (3.6%) | 0 |
|  | Male (yes) | 71 (63.9%) | 0 |
|  | Blood group A (yes) | 46 (41.4%) | 0 |
|  | Blood group AB (yes) | 4 (3.6%) | 0 |
|  | Blood group B (yes) | 8 (7.2%) | 0 |
|  | Blood group 0 (yes) | 53 (47.8%) | 0 |
|  | UC T_KI (yes) | 107 (96.4%) | 0 |
|  | UC I_KI (yes) | 4 (3.6%) | 0 |
|  | Hyperparathyroidism (yes) | 57 (51.4%) | 0 |
|  | Parathyroidectomy (yes) | 11 (9.1%) | 0 |
|  | Pre-transplant dialysis (yes) | 105 (94.6%) | 0 |
|  | Amputation (yes) | 12 (10.81%) | 0 |
|  | Diabetic retinopathy (yes) | 99 (89.19%) | 0 |
|  | Diabetic neuropathy (yes) | 56 (50.5%) | 0 |
|  | Cerebrovascular accident (yes) | 3 (2.7%) | 0 |
|  | Coronary heart disease (yes) | 44 (39.64%) | 0 |
|  | Cardiac infarction (yes) | 8 (7.21%) | 0 |
| **Intra-operative** | **Continuous Variables** | **median (min – max)** | **missing values** |
|  | Cold ischemic period [min (Kidney) | 645 (334 – 1027) | n = 8 (7.2%) |
|  | Warm ischemic period [min] (Kidney) | 25 (11 – 200) | n = 24 (21.6%) |
|  | Cold ischemic period [min] (Pancreas) | 575 (259 – 951) | n = 7 (6.31%) |
| **Post-operative recipient variables** | **Continuous variables** | **median (min – max)** | **missing values** |
|  | Patient survival [years] | 8 (1.1 – 15.5) | 0 |
|  | Graft survival kidney [years] | 7.9 (0 – 15.5) | 0 |
|  | Graft survival pancreas [years] | 6.90 (0 – 14.96) | 0 |
|  | Pulse 1 year after SPK [1/min] | 78 (55 – 107) | n = 13 (11.7%) |
|  | Systolic blood pressure 1 year after SPK [mmHg] | 124.5 (96 – 160) | n = 3 (2.7%) |
|  | Diastolic blood pressure 1 year after SPK [mmHg] | 75 (55 – 100) | n = 3 (2.7%) |
|  | Weight 1 year after SPK [kg] | 71.2 (45 – 116) | n = 3 (2.7%) |
|  | Height 1 year after SPK [cm] | 173 (148 – 193) | 0 |
|  | BMI 1 year after SPK [kg/m²] | 24 (18 – 37) | n = 3 (2.7%) |
|  | GFR 1 year after SPK | 49 (12 – 118) | 0 |
|  | Estimated Creatinine 1 year after SPK [µmol/l] | 121 (64 – 360) | 0 |
|  | Creatinine clearance 1 year after SPK [ml/min] | 60 (18 – 214) | n = 14 (12.6%) |
|  | Calcium 1 year after SPK [mmol/l] | 2.4 (1.75 – 2.81) | 0 |
|  | Potassium 1 year after SPK [mmol]/l | 4.6 (3.5 – 6.5) | 0 |
|  | Sodium 1 year after SPK [mmol/l] | 140 (129 – 146) | 0 |
|  | proteins in urine 1 year after SPK [g/l] | 0 (0.04 – 0.41) | 0 |
|  | HbA1c 1 year after SPK [%] | 6 (4.9 – 10) | n = 1 (0.9%) |
|  | **Binary variables** | **n (% of cohort)** | **missing values** |
|  | Death (yes) | 11 (9.9%) | 0 |
|  | KDIGO I (yes) | 5 (4.5%) | 0 |
|  | KDIGO II (yes) | 25 (22.5%) | 0 |
|  | KDIGO III (yes) | 61 (55%) | 0 |
|  | KDIGO IV (yes) | 18 (16.2%) | 0 |
|  | KDIGO V (yes) | 2 (1.8%) | 0 |
|  | KDIGO ≥ III (yes) | 81 (73%) | 0 |
|  | Re-Dialysis (yes) | 8 (7.21%) | 0 |
|  | HbA1c 1 year after SPK ≤ 6% | 89 (80.01%) | n = 1 (0.9%) |
|  | Increase of HbA1c 1 year after SPK (yes) | 46 (41.4%) | 0 |
|  | Decrease of HbA1c 1 year after SPK (yes) | 38 (34.2%) | 0 |
|  | Insulin therapy after discharge reported (yes) | 14 (12.6%) | 0 |
|  | Insulin therapy 1 year after SPK reported (yes) | 11 (9.91%) | 0 |
|  | Pancreas transplant failure (yes) | 24 (21.82%) | 0 |
|  | Graft pancreatectomy (yes) | 16 (14.41%) | 0 |
|  | Re-Transplantation Pancreas (yes) | 8 (7.2%) | 0 |

**Supplementary Table 1:** Shown is the distribution of pre-, intra- and post-transplant recipient variables determined prior, while and after transplantation (all variables rounded to two decimals). Abbreviations: SPK = simultaneous pancreas-kidney transplantation, HbA1c = Glycosylated Hemoglobin Type A1c, UC I_KI= Urgency code immunized kidney recipient

UC T_KI = Urgency code transplantable kidney recipient

**Title: Development and validation of a prognostic model for kidney function one year after combined pancreas and kidney transplantation using pre-transplant donor and recipient variables**

Journal Name: Langenbeck’s Archives of Surgery

Authors: Katharina S. Zorn, Simon Littbarski , Ysabell Schwager , Alexander Kaltenborn, Jan Beneke, Jill Gwiasda , Thomas Becker, Felix Braun, Benedikt Reichert, Jürgen Klempnauer, Viktor Arelin, Harald Schrem

Corresponding Author: Harald Schrem, MD; [schrem.harald@mh-hannover.de](mailto:schrem.harald@mh-hannover.de)

Affiliations:

Core Facility Quality Management Transplantation, Integrated Research and Treatment Center Transplantation (IFB-Tx), Hannover Medical School, Hannover, Germany

Department of General, Visceral and Transplantation Surgery, Hanover Medical School, Hannover, Germany
