# Supplementary material for: Development and validation of a prognostic model for kidney function 1 year after combined pancreas and kidney transplantation using pre-transplant donor and recipient variables
Source: Langenbecks Arch Surg. 2018 Oct 18;403(7):837–49. doi: 10.1007/s00423-018-1712-z (PMC6244698; doi:10.1007/s00423-018-1712-z)
Supplement: Supplementary file 4 — (DOCX 19 kb) [file 423_2018_1712_MOESM4_ESM.docx]

| **Pre-operative donor variables**  **Training cohort Hannover Medical School** | | |
| --- | --- | --- |
| **Continuous variables** | **median (min – max)** | **missing values** |
| Age [years] | 37 (11 – 51) | 0 |
| Weight [kg] | 68 (40 – 95) | 0 |
| Height [cm] | 170 (155 – 195) | 0 |
| BMI [kg/m²] | 23 (17 – 29) | 0 |
| Number of reported reanimations | 0 (0 – 1) | 0 |
| Time of ventilation [h] | 72.5 (21.1 – 776.5) | n = 2 (1.8%) |
| Duration on ICU [h] | 80.9 (4.7 – 856.8) | n = 32 (28.8%) |
| Creatinine [µmol/l] | 63.6 (34 – 247.5) | 0 |
| GFR | 102.77 (20.12 – 235.15) | 0 |
| Potassium [mmol/l] | 4.1 (2.3 – 6) | 0 |
| Urea [mmol/l] | 3.8 (0.7 – 17.8) | 0 |
| Number of HLA-A mismatches | 1 (0 – 2) | 0 |
| Number of HLA-B mismatches | 2 (0 – 2) | 0 |
| Number of HLA-DR mismatches | 1 (0 – 2) | 0 |
| **Binary variables** | **n (% of cohort)** | **missing values** |
| Male (yes) | 51 (45.9%) | 0 |
| Blood group A (yes) | 46 (41.4%) | 0 |
| Blood group AB (yes) | 4 (3.6%) | 0 |
| Blood group B (yes) | 8 (7.2%) | 0 |
| Blood group 0 (yes) | 53 (47.8%) | 0 |
| Blood group Rhesus positive(yes) | 93 (83.8%) | 0 |
| Cause of death: Trauma (yes) | 4 (7.1%) | n = 55 (49.5%) |
| Cause of death: Subarachnoid hemorrhage (yes) | 12 (21.4%) | n = 55 (49.5%) |
| Cause of death: Respirational (yes) | 1 (1.8%) | n = 55 (49.5%) |
| Cause of death: not otherwise specified (yes) | 1 (1.8%) | n = 55 (49.5%) |
| Cause of death: Cerebral vascular accident bleeding (yes) | 2 (3.6%) | n = 55 (49.5%) |
| Cause of death: Central nervous system tumor (yes) | 1 (1.8%) | n = 55 (49.5%) |
| Cause of death: Central nervous system trauma (yes) | 20 (35.7%) | n = 55 (49.5%) |
| Cause of death: Circulatory (yes) | 2 (3.6%) | n = 55 (49.5%) |
| Cause of death: Cerebral vascular accident not otherwise specified (yes) | 13 (23.2%) | n = 55 (49.5%) |
| HBc Ab (yes) | 1 (0.9%) | 0 |
| CMV IgG (yes) | 54 (48.7%) | 0 |
| Meningitis (yes) | 3 (2.7%) | 0 |
| Heart reported (yes) | 102 (91.9%) | 0 |
| Left lung reported (yes) | 83 (74.7%) | 0 |
| Right lung reported (yes) | 82 (73.9%) | 0 |
| Liver reported (yes) | 111 (100%) | 0 |
| Left kidney reported (yes) | 111 (100%) | 0 |
| Right kidney reported (yes) | 111 (100%) | 0 |
| Intestine reported (yes) | 39 (35.2%) | 0 |
| Cardiac arrest (yes) | 7 (6.3%) | 0 |
| Hypotensive periods (yes) | 12 (10.8%) | 0 |
| Hypertension treatment (yes) | 2 (1.8%) | 0 |
| Smoking (yes) | 29 (26.1%) | 0 |
| Alcohol abuse (yes) | 4 (3.6%) | 0 |
| Urine bacteria (yes) | 1 (0.9%) | 0 |
| Urine cylinders (yes) | 1 (0.9%) | 0 |
| Urine erythocytes (yes) | 21 (18.9%) | 0 |
| Urine glucose (yes) | 3 (2.7%) | 0 |
| Urine leucocytes (yes) | 1 (0.9%) | 0 |
| Urine protein (yes) | 4 (3.6%) | 0 |

**Supplementary Table 2:** Shown is the distribution of pre-operative donor variables determined prior to transplantation (all variables rounded to two decimals). *Abbreviations:* GFR = Glomerular filtration rate, ICU = Intensive Care Unit, HBc Ab = Hepatitis B core antibody, CMV IgG = Cytomegalovirus immunoglobulin G antibody.

**Title: Development and validation of a prognostic model for kidney function one year after combined pancreas and kidney transplantation using pre-transplant donor and recipient variables**

Journal Name: Langenbeck’s Archives of Surgery

Authors: Katharina S. Zorn, Simon Littbarski , Ysabell Schwager , Alexander Kaltenborn, Jan Beneke, Jill Gwiasda , Thomas Becker, Felix Braun, Benedikt Reichert, Jürgen Klempnauer, Viktor Arelin, Harald Schrem

Corresponding Author: Harald Schrem, MD; [schrem.harald@mh-hannover.de](mailto:schrem.harald@mh-hannover.de)

Affiliations:

Core Facility Quality Management Transplantation, Integrated Research and Treatment Center Transplantation (IFB-Tx), Hannover Medical School, Hannover, Germany

Department of General, Visceral and Transplantation Surgery, Hanover Medical School, Hannover, Germany
